# Identifying cancer patients who received palliative care using the SPICT-LIS in medical records: a rule-based algorithm and text-mining technique

**DOI:** 10.1186/s12904-024-01419-1

**Published:** 2024-04-01

**Authors:** Pawita Limsomwong, Thammasin Ingviya, Orapan Fumaneeshoat

**Affiliations:** 1 Department of Family and Preventive Medicine, Prince of Songkla University, Songkhla, 90110 Thailand; 2 Division of Digital Innovation and Data Analytics, Faculty of Medicine, Prince of Songkla University, Hat Yai Campus, Songkhla, 90110 Thailand; 3 Department of Clinical Research and Medical Data Science, Faculty of Medicine, Prince of Songkla University, Songkhla, 90110 Thailand

**Keywords:** Palliative care, cancer patients, Rule-based algorithm, SPICT-LIS criteria, Text-mining techniques

## Abstract

**Background:**

Due to limited numbers of palliative care specialists and/or resources, accessing palliative care remains limited in many low and middle-income countries. Data science methods, such as rule-based algorithms and text mining, have potential to improve palliative care by facilitating analysis of electronic healthcare records. This study aimed to develop and evaluate a rule-based algorithm for identifying cancer patients who may benefit from palliative care based on the Thai version of the Supportive and Palliative Care Indicators for a Low-Income Setting (SPICT-LIS) criteria.

**Methods:**

The medical records of 14,363 cancer patients aged 18 years and older, diagnosed between 2016 and 2020 at Songklanagarind Hospital, were analyzed. Two rule-based algorithms, strict and relaxed, were designed to identify key SPICT-LIS indicators in the electronic medical records using tokenization and sentiment analysis. The inter-rater reliability between these two algorithms and palliative care physicians was assessed using percentage agreement and Cohen’s kappa coefficient. Additionally, factors associated with patients might be given palliative care as they will benefit from it were examined.

**Results:**

The strict rule-based algorithm demonstrated a high degree of accuracy, with 95% agreement and Cohen’s kappa coefficient of 0.83. In contrast, the relaxed rule-based algorithm demonstrated a lower agreement (71% agreement and Cohen’s kappa of 0.16). Advanced-stage cancer with symptoms such as pain, dyspnea, edema, delirium, xerostomia, and anorexia were identified as significant predictors of potentially benefiting from palliative care.

**Conclusion:**

The integration of rule-based algorithms with electronic medical records offers a promising method for enhancing the timely and accurate identification of patients with cancer might benefit from palliative care.

**Supplementary Information:**

The online version contains supplementary material available at https://doi.org/10.1186/s12904-024-01419-1.

## Background

Globally, approximately 40 million individuals require palliative care each year, with 78% living in low- and middle-income countries where palliative care resources, including home ventilators, are limited [1]. Only 14% of these patients are estimated to receive appropriate palliative care [1]. Several factors have resulted in the limited access to palliative care in these countries. Moreover, resources including palliative care specialists, devices such as oxygen generators and syringe drivers are limited [2]. In developing countries, including Thailand, there are also few hospice services available [3], which further exacerbates the accessibility challenges. One suggestion to help alleviate such accessibility challenges is priority screening, so only patients who might benefit from palliative care are offered the service. Experts have challenged this issue by establishing various scoring systems and criteria to help prioritize and select patients [4].

Several tools have been developed to help physicians and/or healthcare teams assess patients who should receive palliative care [5]. The Supportive and Palliative Instrument Indicators Tool (SPICT) [6] is the most common tool used to help healthcare professionals identify patients with advanced life-limiting conditions who may benefit from a holistic palliative care program. The SPICT was first published in 2014 and has been used in over 30 countries [6]. A modified version of the SPICT, the Supportive and Palliative Care Indicators for a Low-Income Setting (SPICT-LIS), was created in 2019 for use in low-income countries [6] .

In 2021, the SPICT-LIS was translated into Thai, with cross-cultural validation by Sripaew et al. and subsequently tested using real-world data in a retrospective study by Fumaneeshoat et al. [7] using the Thai-translated SPICT-LIS to identify cancer patients who may have benefited from palliative care in Thailand. They found that 7.8% of 9,990 patients with cancer might have qualified for palliative care [8]. However, it is challenging to evaluate all cancer patients using an instrument such as the SPICT-LIS to evaluate which patients would benefit from palliative care in real life due to limited resources, healthcare providers, and knowledge. If a simpler, more user-friendly screening tool could be developed, it would be easier for healthcare professionals to identify patients might benefit from palliative care.

Appropriate text-mining techniques and text-based artificial intelligence such as Natural Language Processing could potentially play a very useful role in modern healthcare with its voluminous electronic medical records owning to their ability to extract information from unstructured clinical text data such as medical records and physician notes [9]. These techniques, which are powered by algorithms, are designed to’ efficiently process and analyze large volumes of textual data [10].

Therefore, this study aimed to create a rule-based algorithm based on regular expressions and sentiment analysis to identify patients who might be benefit from palliative care based on the Thai SPICT-LIS criteria from electronic medical records. The factors and characteristics of patients recommended for palliative care by palliative care specialists were used to develop and adjust the rule-based algorithm to improve the accuracy of the model.

## Materials and methods

### Study design and setting

The electronic medical records, including electronic doctor’s notes (eDN) and patient characteristics were extracted from Songklanagarind Hospital, the biggest hospital in Southern Thailand, database. The data scientists of the Division of Digital Innovation and Data Analytics (DIDA), Faculty of Medicine, Prince of Songkla University, supervised by Dr. Ingviya, randomly selected 100 inpatients diagnosed with cancer confirmed by the Cancer Registry and prepared their eDNs and patient characteristics to be reviewed. Two palliative care physicians independently reviewed the medical records of the 100 randomly selected cancer patients between February and June 2022. The records were reviewed using the Thai version of the SPICT-LIS to determine whether palliative care would have been beneficial to the patients.

### Data source

The data of the study cancer patients were retrieved from the Cancer Registry of Songklanagarind Hospital, and further documents were queried from the hospital inpatient department data (IPD) prepared by DIDA as mentioned above. The eDN, patient’s characteristics and vital signs were extracted and stored using the PostgreSQL Relational Database Management System on a physical server in the DIDA Data Center. The querying and merging of text data were done though the PostgreSQL.

### Inclusion/ exclusion criteria

All cancer inpatients aged 18 years or older diagnosed with cancer at Songklanagarind Hospital using the International Classification of Diseases and Related Health Problems 10th Revision Thai Modification (ICD-10 TM) [11] and the International Classification of Disease for Oncology (ICD-O) [12] from 2016 to 2020 were included in the initial study sample. Patients who had a first admission digital note of ≤ 1,000 words following their cancer diagnosis were excluded from the study to ensure that the study records had an adequate amount of the data required to assess the patients using the Thai SPICT-LIS criteria. The patient characteristics data extracted included birth date, sex, religion, ICD-10 and ICD-O, and cancer staging.

### Data management and algorithm development

#### Training set

To create an initial training dataset, two palliative care physicians reviewed the whole records of 100 randomly selected patients and assessed if the patients had any of the six general indicators suggesting that they might benefit from palliative care, which were coded as 1 or 0 for patients who might or might not benefit, respectively. When there was disagreement between the two specialists, a consensus was reached by a face-to-face discussion.

#### Text-mining models

Text-mining models were created to extract essential data from standard language text in both the Thai and English languages eDN data via text mining by using a sequence of characters that formed a search pattern called a tokenization technique [13] (regular expression) [14] with the ‘LexTo’ package, a package enabling tokenization of the Thai Language in the R program.

#### Sentiment analysis

Sentiment analysis involves classifying data into categories like positive or negative [15]. For instance, the word “pain” might be labeled as negative, whereas the phrase “no pain” could be considered positive. Text segments in the code were passed directly as input to the model. In this study, the sentiment analysis model was trained to categorize the sentiment of a given text into two groups, patients who might be benefit from palliative care and those who might not.

#### Data dictionaries

A data dictionary encompassing a range of mixed Thai and English words/phrases was created using tokenization and sentiment analysis to classify patients into 2 groups based on whether they satisfied any of the six Thai SPICT-LIS general indicators or not. In general, words/phrases and/or sentences indicating symptoms and patient history were used to determine if the patients had presented with any of the six general indicators. The classification and extraction of each general indicator was performed on the physicians’ free-text comments using the mixed language data dictionary. For international readers of our paper, we translated the Thai words/phrases/sentences in the data dictionary to universally understood English terminology presented side by side with the Thai corresponding words/sentences as detailed in Table S1.

#### Rule-based algorithms

Two Rule-based algorithms were created based on Regular expression, Tokenization and Sentiment Analysis using the R Program version 4.0.3 (R Core Team, Austria) from the whole records written in a mixture of Thai and English words/phrases/sentences.

Strict and relaxed rule-based algorithms were used in this study. Strict-rule-based criteria were defined using a stringent set of criteria for identifying each indicator. The strict algorithm was characterized by its focus on using explicit and well-defined terms, which could have led to fewer cases meeting the criteria. In contrast, the relaxed rule-based algorithm used a more flexible approach characterized by its inclusiveness in considering a variety of factors that could have indicated the presence of the condition, which could have resulted in a larger number of identified cases. For example, in the strict rule-based criteria of the Thai SPICT-LIS algorithm, only ‘significant weight loss’ was included, while in the relaxed rule-based approach, additional keywords such as ‘weight loss,’ ‘underweight,’ ‘hyposthenic build,’ and ‘thinner’ were also considered alongside significant weight loss.

### Outcome measurements

The main outcome was done to find the algorithm correctly identified patients who might benefit from palliative care as indicated by the SPICT-LIS. The instrument was originally back-translated into Thai by Sripaew et al., following the WHO guidelines for the systematic adaptation of tools, and was then found to provide consistent responses with good agreement among general practitioners, with a Fleiss-Kappa of 0.93 (0.76–1.00) [7]. The six indicators of the Thai SPICT-LIS are as follows: Indicator 1: performance status is poor or deteriorating, best available treatment has limited effect; Indicator 2: depends on others for care due to increasing physical and/or mental health problems; Indicator 3: the individual’s carer requires more help and support; Indicator 4: the individual experienced significant weight loss over the last few months or remains underweight; Indicator 5: persistent symptoms despite receiving the best available treatment for underlying condition(s) and is unable to access treatment; and Indicator 6: the individual (or family) asks for palliative care and chooses to reduce, stop, or not have treatment or wishes to focus on quality of life. Patients who would possibly benefit from palliative care were those who met at least two general indicators and at least one clinical indicator [7, 16, 17]. Patients who met these same criteria were defined as “should be offer palliative care as they could benefit from it” and the others were defined as “should not be offered” palliative care.do not meet the indicators for being offered palliative care at this time.

### Inter-rater reliability

Percentage agreement and kappa statistics were used to measure the inter-rater reliability between the physicians and the strict and relaxed rule-based algorithms. Cohen’s kappa was interpreted as follows: a value above 0.7 indicates good agreement; values between 0.4 and 0.7 indicate moderate agreement; and values below 0.4 indicate poor agreement [18].

### Prevalence and factor association

The number of patients with cancer who should be given palliative care as they will be benefit from it was compared between palliative care specialists and both strict and relaxed rule-based algorithms. Descriptive statistics and Fisher’s exact tests were used to compare the characteristics of patients with cancer who should be given palliative care with those of patients who should not. Factors associated with patients with cancer who required palliative care were assessed using Fisher’s exact test and multiple logistic regression analysis. Multiple logistic regression analysis to assess the factors associated with requiring palliative care including age, sex, cancer type, cancer stage, and patient symptoms such as pain, dyspnea, anorexia, edema, dysphagia, ascites, and xerostomia. A *p*-value of less than 0.05 was considered statistically significant.

## Results

**Fig. 1:**
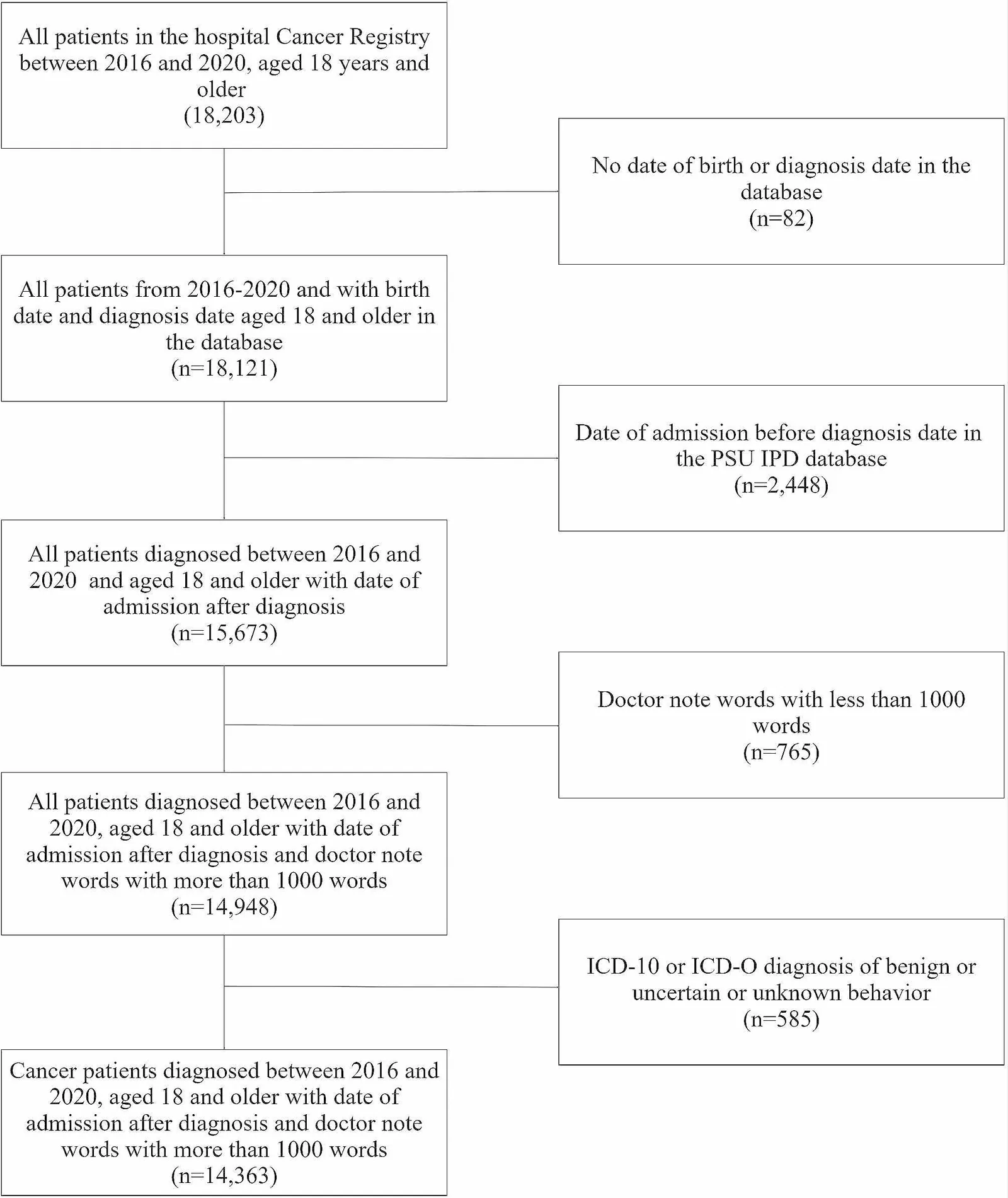
Participant flow chart

A total of 18,203 patients were enrolled in the Cancer Registry of Songklanagarind Hospital during the study period, of whom 2,448 patients whose admission dates preceded their cancer diagnosis dates were excluded. Additionally, 765 patients whose doctors’ notes contained fewer than 1,000 words, and 585 patients diagnosed with benign masses or masses of unknown behavior were also excluded. The final analysis included a total of 14,363 patients as presented in Fig. 1.

In the training dataset, comprising the admission notes of 100 patients, the comparison between rule-based and human assessment by palliative care physicians showed the proportion of patients meeting the SPICT-LIS criteria. The strict rule-based algorithm showed a high percentage agreement of 95% with the trained physicians, with a Cohen’s kappa coefficient of 0.83 (0.67–0.99), indicating strong concordance. While the relaxed rule-based algorithm showed a percentage agreement of 71% and a Cohen’s kappa coefficient of 0.16 (0.02–0.30), indicating lower agreement levels, as detailed in Table S2.

From 2016 to 2020, 14,363 cancer patients, met the study criteria. Approximately 65% of the patients were aged < 65 years. The years with male-to-female ratio was approximately 1:1. The most common types of cancer were gastrointestinal and gynecological cancers, followed by breast cancer. Approximately 45% of the patients had stage 3 or 4 cancer at the time of diagnosis (Table 1).

**Table 1:** Participants’ sociodemographic and clinical characteristics (*n* = 14,363)

Factor	n% (14,363)	Relaxed rule-based criteria			Strict rule-based criteria		
		No	Yes	*p*-value	No	Yes	*p*-value
Age				0.017			< 0.001
< 65	9531 (66.4)	6852 (66.8)	2119 (64.2)		7751 (66.7)	990 (62.1)	
≥ 65	4832 (33.6)	3398 (33.2)	1163 (35.4)		3876 (33.3)	603 (37.9)	
Sex				< 0.001			0.005
Female	7742 (53.9)	5735 (56)	1556 (47.4)		6375 (54.8)	814 (51.1)	
Male	6621 (46.1)	4515 (44)	1726 (52.6)		5252 (45.2)	779 (48.9)	
Religion				0.783			0.056
Buddhist	12,236 (85.2)	8738 (85.2)	2782 (84.8)		9934 (85.4)	1325 (83.2)	
Islam	2058 (14.3)	1463 (14.3)	483 (14.7)		1637 (14.1)	260 (16.3)	
Other	69 (0.5)	49 (0.5)	17 (0.5)		56 (0.5)	8 (0.5)	
Stage				< 0.001			< 0.001
1	1828 (12.7)	1532(20.2)	204 (8.4)		1626 (18.9)	87 (7.8)	
2	2163 (15.1)	1705(22.5)	331(13.6)		1850 (21.5)	156 (14)	
3	2822 (19.6)	2108(27.8)	570 (23.5)		2373 (27.5)	231 (20.8)	
4	3714 (26.6)	2242 (29.6)	1324 (54.5)		2766 (32.1)	639 (57.4)	
Disease site				< 0.001			< 0.001
Breast	1710 (11.9)	1323(12.9)	282 (8.6)		1437 (12.4)	159 (10)	
Endocrine	510 (3.6)	366 (3.6)	78 (2.4)		405 (3.5)	33 (2.1)	
Gastrointestinal	3786 (26.4)	2748 (26.8)	725 (22.1)		3102 (26.7)	304 (19.1)	
Gynecological	2538 (17.7)	2007 (19.6)	420 (12.8)		2218 (19.1)	176 (11)	
Hematological	1007 (7.0)	661 (6.4)	324 (9.9)		782 (6.7)	194 (12.2)	
Head and neck	1426 (9.9)	975 (9.5)	383 (11.7)		1120 (9.6)	97 (6.1)	
Male genitals	659 (4.6)	542 (5.3)	75 (2.3)		576 (5)	38 (2.4)	
Mesothelial and soft tissue	258 (1.8)	174 (1.7)	78 (2.4)		209 (1.8)	41 (2.6)	
Other	294 (2.0)	126 (1.2)	159 (4.8)		149 (1.3)	125 (7.8)	
Respiratory and intrathoracic organs	1275 (8.9)	664 (6.3)	593 (18.1)		287 (2.5)	63 (4)	
Skin	367 (2.6)	261 (2.5)	94 (2.9)		453 (3.9)	38 (2.4)	
Urinary tract	533 (3.7)	423 (4.1)	71 (2.2)				
Pain				< 0.001			< 0.001
No		5317 (51.9)	948 (28.9)		5648 (48.6)	510 (32)	
Yes		4933 (48.1)	2334 (71.1)		5979 (51.4)	1083 (68)	
Dyspnea				< 0.001			< 0.001
No		9167 (89.4)	2050 (62.5)		9964 (85.7)	964 (60.5)	
Yes		1083 (10.6)	1232 (37.5)		1663 (14.3)	629 (39.5)	
Edema				< 0.001			< 0.001
No		9766 (95.3)	2886 (87.9)		10,983 (94.5)	1370 (86)	
Yes		484 (4.7)	396 (12.1)		644 (5.5)	223 (14)	
Delirium				< 0.001			< 0.001
No		10,216 (99.7)	3230 (98.4)		11,581 (99.6)	1554 (97.6)	
Yes		34 (0.3)	52 (1.6)		46 (0.4)	39 (2.4)	
Xerostomia				< 0.001			< 0.001
No		10,143 (99)	3168 (96.5)		11,490 (98.8)	1510 (94.8)	
Yes		107 (1)	114 (3.5)		137 (1.2)	83 (5.2)	
Ascites				0.103			0.033
No		9296 (90.7)	2945 (89.7)		10,531 (90.6)	1416 (88.9)	
Yes		954 (9.3)	337 (10.3)		1096 (9.4)	177 (11.1)	
Dysphagia				< 0.001			0.035
No		9698 (94.6)	2904 (88.5)		10,836 (93.2)	1507 (94.6)	
Yes		552 (5.4)	378 (11.5)		791 (6.8)	86 (5.4)	
Anorexia				< 0.001			< 0.001
No		9614 (93.8)	2756 (84)		10,764 (92.6)	1332 (83.6)	
Yes		636(6.2)	526 (16)		863 (7.4)	261 (16.4)	

**Table 2:** Comparison of patients meeting one or more SPICT-LIS criteria by the text-mining algorithms

Indicators	Relaxed-rule-based algorithm (N (%))	Strict-rule-based algorithm (N (%))
Indicator 1: performance status is poor or deteriorating, best available treatment has limited effect	3494 (24.3)	2003 (13.9)
Indicator 2: depends on others for care due to increasing physical and/or mental health problems	3412 (23.8)	1287 (9.0)
Indicator 3: the individual’s carer requires more help and support	8 (0.1)	8 (0.1)
Indicator 4: the individual experienced significant weight loss over the last few months, or remains underweight	2533 (17.6)	1969 (13.7)
Indicator 5: persistent symptoms despite receiving the best available treatment for underlying condition(s); is unable to access treatment	39 (0.3)	39 (0.3)
Indicator 6: the individual (or family) asks for palliative care; chooses to reduce, stop, or not have treatment; or wishes to focus on quality of life	1126 (7.8)	1039 (7.2)
Met criteria	3282 (22.9)	1593 (11.1)

Table 2 presents the number of patients with cancer who met the criteria for each indicator classified between the relaxed rule-based algorithm and the strict rule-based algorithm. Regarding the number of patients with cancer who could possibly benefit from palliative care according to these different algorithms, of the 14,363 identified study cancer patients in Songklanagarind Hospital, the strict rule-based algorithm resulted in 11.1per 100 hospitalized patients with cancer, while the relaxed rule-based algorithm resulted in 22.9 per 100 hospitalized patients with cancer.

Univariate analysis indicated that the number of patients with cancer eligible for palliative care increased with increasing age, higher cancer stage and certain specific sites of primary cancer, and symptoms such as pain, dyspnea, edema, anorexia, xerostomia, delirium, ascites, and dysphagia, which were associated with higher odds of patients with cancer like to benefit from palliative care (see table S3).

The investigation into factors associated with a high likelihood of requiring palliative care utilized two different algorithms: a relaxed rule-based algorithm and a strict rule-based algorithm. In the relaxed approach, sex (OR = 1.25, 95% CI: 1.08–1.43), specific cancer sites, cancer stages, and various symptoms were associated with higher probabilities of the patient with cancer who met SPICT-LIS criteria Notably, the agreement rate between relaxed rule-based and human assessment by palliative care physician was 71%, indicating moderate concordance.

On the other hand, the strict rule-based algorithm found older age (OR = 1.18,95% CI: 1.02–1.37), specific cancer sites, cancer stages, and symptoms, resulting in a high agreement rate between the strict rule-based algorithm and human assessment by palliative care physicians of 95% as shown in Table 3.

**Table 3:** Multiple logistic regression analysis for the prevalence of study cancer patients meeting the SPICT-LIS criteria

Factor		Relaxed rule-based criteria		Strict rule-based criteria	
		OR (95% CI)	*p*-value	OR (95% CI)	*p*-value
Age	< 65	-	0.182	-	0.003
	≥ 65	1.08 (0.96–1.21)		1.18 (1.02–1.37)	
Sex	Female	-	0.002	-	0.937
	Male	1.25 (1.08–1.43)		1.00 (0.84–1.2)	
Disease	Breast	Ref	< 0.001	ref	< 0.001
	Endocrine	0.93 (0.53–1.58)		0.740 (0.32–1.38)	
	Gastrointestinal	0.78 (0.63–0.97)		0.60 (0.46–0.79)	
	Gynecological	0.78 (0.68–1.02)		0.57 (0.44–0.74)	
	Hematological	0.81 (0.62–1.07)		0.93 (0.67–1.28)	
	Head and neck	0.87 (0.68–1.11)		0.47 (0.33–0.65)	
	Male genitals	0.53 (0.35–0.80)		0.58 (0.35–0.96)	
	Mesothelial and soft tissue	1.78 (1.11–2.83)		1.73 (0.99–2.93)	
	Other	1.01 (0.57–1.73)		1.38 (0.72–2.54)	
	Respiratory and intrathoracic organs	1.51 (1.19–1.91)		1.14 (0.86–1.52)	
	Skin	2.48 (1.52–3.98)		3.21 (1.87–5.38)	
	Urinary tract	0.60 (0.39–0.91)		0.55 (0.31–0.93)	
Stage	1	ref	< 0.001	ref	< 0.001
	2	1.33 (1.08–1.63)		1.38 (1.04–1.83)	
	3	1.65 (1.36–1.99)		1.58 (1.21–2.07)	
	4	2.76 (2.29–3.32)		3.00 (2.34–3.91)	
Pain	No		< 0.001		< 0.001
	Yes	2.38 (2.13–2.66)		1.69 (1.46–1.96)	
Dyspnea	No		< 0.001		< 0.001
	Yes	4.30 (3.79–4.88)		3.11 (2.67–3.63)	
Edema	No		< 0.001		< 0.001
	Yes	1.63 (1.33–1.99)		1.61 (1.28–2.01)	
Delirium	No		< 0.001		< 0.001
	Yes	3.17 (1.70–5.88)		3.52 (1.80–6.63)	
Xerostomia	No		0.005		< 0.001
	Yes	1.68 (1.17–2.42)		2.50 (1.73–3.60)	
Ascites	No		0.035		0.459
	Yes	0.81 (0.67–0.98)		0.91 (0.71–1.16)	
Dysphagia	No		< 0.001		0.138
	Yes	2.46 (2.06–2.94)		0.81 (0.61–1.06)	
Anorexia	No		< 0.001		< 0.001
	Yes	2.23 (1.90–2.62)		2.00 (1.66–2.43)	

## Discussion

### The main findings

This study used a rule-based algorithm based on text tokenization and sentiment analysis to identify patients with cancer who should be given palliative care as they will benefit from it according to the SPICT-LIS criteria. Due to healthcare resource limitations, especially in palliative care, in low- and middle-income [19] countries such as Thailand [20], the understanding of the care process and access to palliative care remains limited [21]. From this study, the two rule-based algorithms of the SPICT-LIS into electronic medical records or hospital information systems will assist physicians in the early detection of patients who may benefit from palliative care services. The results of the study highlight the potential use and effectiveness of rule-based algorithms for identifying palliative care cases.

The study designed two rule-based algorithms to identify the key SPICT-LIS indicators in medical records. This algorithm was developed focusing on patients with cancer. The rules were formulated based on clinical guidelines and expert knowledge, allowing the algorithm to recognize related terms and phrases related to the SPICT-LIS criteria [7].

These findings have significant implications for healthcare providers and researchers. Rule-based algorithms show a promising ability to assist in identifying patients who may benefit from palliative care. Therefore, it is a potential tool for improving patient access to palliative care.

### Relaxed and strict rule-based algorithms

Two approaches relaxed and strict rule-based algorithms, were implemented and compared. This comparison aimed to assess the accuracy and efficiency of a rule-based algorithm for evaluating palliative care candidates among patients with cancer. The strict approach applied stringent criteria to identify patients who met at least one of the SPICT-LIS indicators. The results showed that the strict rule-based algorithm demonstrated a high degree of specificity in identifying cancer patients who might benefit from palliative care, or in other words, showed a low false positive rate. However, this study acknowledged concerns regarding potentially missed cases that may occur with the strict rule-based criteria, and therefore a relaxed rule-based algorithm that identified a broader range of cases, but with a certain false-positive rate, may be more appropriate for use as a primary screening tool. Specifically, if the relaxed rule-based algorithm identifies a patient as positive, while the strict rule-based algorithm does not, a physician should be involved to evaluate these patients to ensure the coverage of all patients who may benefit from palliative care services.

### The factors associated with patients who might benefit from palliative care

The factors associated with patients who might benefit from palliative care, as determined by the rule-based algorithm, included advanced-stage cancer and symptoms such as pain, dyspnea, edema, delirium, xerostomia, and anorexia. Most of the associated factors were consistent with previous studies, in which cases who should receive palliative care were determined based on physician judgment [22, 23].

Advanced-stage cancer has been reported as a significant predictor of patients requiring palliative care in various studies [24]. Patients diagnosed at later stages of cancer often experience more severe symptoms and complications, making them potential candidates for palliative interventions [25]. Pain is a prominent concern in palliative care utilization [26]. Cancer-related pain can be debilitating, making effective pain management an important aspect of palliative care [27]. Dyspnea and other symptoms are often observed in patients with advanced cancer and are important indicators for receiving palliative care [28, 29]. Additionally, edema [30], delirium [31], xerostomia, and anorexia [29] are also positive predictors of palliative care utilization in patients with cancer. These symptoms contribute to the complex symptom burden that palliative care aims to alleviate, emphasizing the importance of early and comprehensive symptom assessments for appropriate palliative care interventions [29, 32].

### Limitations

This study had several limitations. First, there is wide variability in clinical documentation and terminology found in medical records. Rule-based algorithms can make them less adaptable to the diverse languages and documentation practices prevalent in various healthcare institutions. Second, the accuracy of the algorithm results may be influenced by the quality and completeness of the medical records; incorrect or incomplete information can result in false positives and negatives.

### Suggestions

To improve the application of text mining and rule-based algorithms in palliative care identification, several key future directions should be explored. First, algorithm rules should be refined to accommodate diverse clinical contexts and terminologies in collaboration with healthcare professionals to improve accuracy. Second, the development of healthcare systems in low-to-middle-income countries is technologically limited, to improve the situation, we strongly support the implementation of a country-wide health information system should be developed. The use of algorithms such as described above could facilitate the improved use of health records in identifying people who might benefit from palliative care, or other emerging treatments.

Future research with the large dataset should focus on more advance Natural Language Processing techniques including the uses of deep Bidirectional Encoder Representations from Transformers or generative artificial intelligence for more accurate classification of patients who might be benefit from palliative care.

## Conclusion

This study demonstrated the potential of rule-based algorithms and text-mining techniques using medical records in identifying patients with cancer who will benefit from palliative care based on the SPICT-LIS criteria. This approach offers a promising solution to improve the timeliness and accuracy of palliative care case identification.

## Electronic supplementary material

Below is the link to the electronic supplementary material.

Supplementary Material 1

## Data Availability

The datasets used and/or analyzed during the current study are available from the corresponding author on reasonable request.

## Abbreviations

DIDA: Digital Innovation and Data Analytics
ICD-10 TM: International Classification of Diseases and Related Health Problems 10th Revision Thai Modification
ICD-O: International Classification of Diseases for Oncology
IPD: In-Patient Department
SPICT-LIS: Supportive and Palliative Care Indicators for a Low-Income Setting
