# Supplementary material for: Identifying cancer patients who received palliative care using the SPICT-LIS in medical records: a rule-based algorithm and text-mining technique
Source: BMC Palliat Care. 2024 Apr 1;23:83. doi: 10.1186/s12904-024-01419-1 (PMC10983682; doi:10.1186/s12904-024-01419-1)
Supplement: Supplementary file 1 — Supplementary Material 1 [file 12904_2024_1419_MOESM1_ESM.docx]

Supplementary Materials: Identifying Cancer Patients Who Received Palliative Care Using the SPICT-LIS in Medical Records: A Rule-Based Algorithm and Text-Mining Technique

Table 1. Text groups in relaxed and strict rule-based algorithms

| Indicator | Relaxed rule-based | | Strict rule-based | |
| --- | --- | --- | --- | --- |
|  | Thai | Translated to English | Thai | Translated to English |
| Indicator 1: performance status is poor or deteriorating, best available treatment has limited effect | ติดเตียง  ขยับตัวไม่ได้  เดินไม่ได้  อ่อนแรง  อัมพาต  ไม่สามารถเคลื่อนไหวแขนขาได้  นั่งเป็นส่วนใหญ่  ใช้อุปกรณ์ช่วยเดิน  อ่อนแรง  เพลีย  ขณะเดินต้องมีเวลพัก  เหนื่อยมากขึ้น, หายใจลำบาก  ไม่สามารถนอนได้  กินอาหารไม่ได้  ซึม  หลับเป็นส่วนใหญ่  ตอบสนองช้า  ตอบคำถามช้า  มีประวัติซึมลง  มีประวัติหัวใจหยุดเต้น  ต้องการความช่วยเหลือจากผู้อื่น  ญาติจัดเตรียมอาหารให้  ไม่สามารถกินอาหารทางปากได้  มีปัญหาเรื่องการกลืน  กลืนเจ็บ  กลืนติด  ปวดมากขึ้น  อาการแย่ลง  ECOG มากกว่า 3 | Bed-ridden  Cannot turn him/herself over  Cannot walk  Weakness  Paralysis  Inability to move limbs  Mostly sitting  Walking aid  Fatigue  Have to stop often when walking  Progressive dyspnea, shortness of breath, chest tightness, difficulty breathing  Unable to lie down  Unable to receive food  Cannot work  Unable to wake up  Loss of consciousness  Sleeping more frequently  Slow response to commands  Slow in responding to questions  Experiencing loss of consciousness  Post-arrest  Requires assistance with daily activities  Relatives provide meals  Unable to eat using their mouth  Difficulty swallowing  Pain when swallowing  Dysphagia  Symptoms worsening  Condition deteriorating  Condition not getting better  Increasing pain  Severe pain  Intense pain  ECOG ≥3 | ติดเตียง  ขยับตัวไม่ได้  เดินไม่ได้  อ่อนแรง  อัมพาต  ไม่สามารถเคลื่อนไหวแขนขาได้  นั่งเป็นส่วนใหญ่  กินอาหารไม่ได้  ไม่สามารถกินอาหารทางปากได้  ซึม  หลับเป็นส่วนใหญ่  ตอบสนองช้า  ตอบคำถามช้า  ต้องการความช่วยเหลือจากผู้อื่น  ญาติจัดเตรียมอาหารให้  อาการแย่ลง  ECOG มากกว่า 3 | Bed-ridden  Cannot turn him/herself over  Cannot walk  Weakness  Paralysis  Inability to move limbs  Mostly sitting  Unable to receive food  Unable to eat using their mouth  Unable to wake up  Loss of consciousness  Sleeping more frequently  Slow response to commands  Slow in responding to questions  Requires assistance with daily activities  Relatives provide meals  Symptoms worsening  Condition deteriorating  ECOG ≥3 |
| Indicator 2: depends on others for care due to increasing physical and/or mental health problems | ไม่สามารถเคลื่อนไหวเองได้  เดินไม่ได้  อ่อนแรง  อัมพาต  มีปัญหาเรื่องการเคลื่อนไหวแขนขา  นั่งเป็นส่วนใหญ่  ใช้อุปกรณ์ช่วยเดิน  เพลีย  ขณะเดินต้องพัก  เหนื่อยมากขึ้น  หายใจไม่สุด  หายใจลำบาก  ไม่สามารถนอนได้  ซึม  หลับเป็นส่วนใหญ่  ตอบสนองช้า  ตอบคำถามช้า  มีประวัติซึมลง  มีประวัติหัวใจหยุดเต้น  เหนื่อยมากขึ้นเวลาขยับตัว  สับสน  ไม่สามารถกินอาหารได้  ไม่สามารถกินอาหารทางปากได้  มีปัญหาเรื่องการกลืน  กลืนติด  ต้องการความช่วยเหลือจากผู้อื่น  ญาติต้องเตรียมอาหารให้  มีคนจัดยาให้  อาการแย่ลง  ใช้ oxygen | Cannot turn him/herself over  Cannot walk  Weakness  Paralysis  Inability to move limbs  Mostly sitting  Walking aid  Fatigue  Have to stop often when walking  Progressive dyspnea  Shortness of breath  Chest tightness  Difficulty breathing  Unable to lie down  Loss of consciousness  Sleeping more frequently  Unable to wake up  Slow response to commands  Slow in responding to questions  Experiencing loss of consciousness  Post-arrest  Getting more tired when moving  Confused  Unable to receive food  Unable to eat using their mouths  Unable to swallow  Difficulty swallowing  Pain when swallowing  Dysphagia  Requires assistance with daily activities  Relatives provide meals  Feeding  Providing meals  Assisted by someone for support  Arranging medication  Symptoms worsening  Condition deteriorating  Use of an oxygen cannula | ไม่สามารถเคลื่อนไหวเองได้  อ่อนแรง  อัมพาต  นั่งเป็นส่วนใหญ่  ไม่สามารถกินอาหารได้  ไม่สามารถกินอาหารทางปากได้  ซึม  หลับเป็นส่วนใหญ่  ตอบสนองช้า  ตอบคำถามช้า  เหนื่อยมากขึ้นเวลาขยับตัว  สับสน  ต้องการความช่วยเหลือจากผู้อื่น  ญาติต้องเตรียมอาหารให้  มีคนจัดยาให้ | Cannot turn him/herself over  Weakness  Paralysis  Mostly sitting  Unable to receive food  Unable to eat using his/her mouth  Unable to wake up  Loss of consciousness  Sleeping more frequently  Slow response to commands  Slow in responding to questions  Getting more tired when moving  Confused  Requires assistance with daily activities  Relatives provide meals  Assisted by someone for support  Arranging medication |
| Indicator 3: the individual’s carer requires more help and support | ญาติกังวล  ยุติการรักษา | Relatives are anxious  Relatives are worried  Termination of treatment  Cessation of treatment | ยุติการรักษา | Termination of treatment  Cessation of treatment |
| Indicator 4: the individual experienced significant weight loss over the last few months, or remains underweight | น้ำหนักลด  ผอม | Weight loss  Cachexic  Thinner  Slenderizing  Hyposthenic build | น้ำหนักลดลงอย่างมีนัยสำคัญ | Significant weight loss |
| Indicator 5: persistent symptoms despite receiving the best available treatment for underlying condition(s); is unable to access treatment | มีปัญหาเรื่องการเดินทาง  ปัญหาด้านค่าใช้จ่าย  บ้านห่างไกล  ไม่สามารถจ่ายได้  ญาติมารับยาแทน  ญาติมาแทน | Travel issues  Financial hardship  Expense problems  Cost issues  Far from home  Patients unable to pay for themselves  Indigence  Poverty  Relatives arrive as substitute  Relatives pick up medicine | มีปัญหาเรื่องการเดินทาง  ปัญหาด้านค่าใช้จ่าย  บ้านห่างไกล  ไม่สามารถจ่ายได้ | Travel issues  Financial hardship  Expense problems  Cost issues  Far from home  Patients unable to pay for themselves  Indigence  Poverty |
| Indicator 6: the individual (or family) asks for palliative care; chooses to reduce, stop, or not have treatment; or wishes to focus on quality of life | ยุติการรักษา  Palliative  ไม่ขอรับการช่วยชีวิต  No CPR  No ETT  ไม่ใส่สาย  หยุดการรักษา  ไม่ขอรับการรักษา  ดูแลแบบประคับประคอง  Best supportive care  รักษาตามอาการ/ Conservative treatment  ญาติและผู้ป่วยปฏิเสธการใส่ท่อช่วยหายใจ | Termination of treatment  Palliative  Do not resuscitate  No cardiopulmonary resuscitation  No feeding tubes  No insertion of a tube  No endotracheal tube  Stop treatment  Discontinue treatment  Best supportive care  Conservative treatment  Relatives and patients refuse intubation for assisted respiration | ยุติการรักษา  Palliative  ไม่ขอรับการช่วยชีวิต  No CPR  No ETT  หยุดการรักษา  ไม่ขอรับการรักษา  ดูแลแบบประคับประคอง  Best supportive care  ญาติและผู้ป่วยปฏิเสธการใส่ท่อช่วยหายใจ | Termination of treatment  Palliative  Do not resuscitate  No cardiopulmonary resuscitation  No endotracheal tubes  Stop treatment  Discontinue treatment  Best supportive care  Relatives and patients refuse intubation for assisted respiration |

Table 2. Comparing Palliative Care Utilization Among Cancer Patients

| SPICT-LIS | | Relaxed rule-based criteria | | Strict rule-based criteria | |
| --- | --- | --- | --- | --- | --- |
|  |  | Met criteria | Did not meet criteria | Met criteria | Did not meet criteria |
| Palliative care physician | Met criteria | 22 | 3 | 17 | 5 |
|  | Did not meet criteria | 26 | 49 | 0 | 78 |
|  | Percentage agreement (%) | 71 | | 95 | |
|  | Cohen’s kappa | 0.16 (0.02–0.30) | | 0.83 (0.67–0.99) | |

Table 3. Univariate analysis of the prevalence of patients with cancer meeting the SPICT-LIS criteria

| Factor | | Relaxed Rule-Based | | Strict Rule-Based | |
| --- | --- | --- | --- | --- | --- |
|  |  | OR (95% CI) | p-value | OR (95% CI) | p-value |
| Age | <65 | - | <0.001 | - | <0.001 |
|  | ≥65 | 1.11 (1.02–1.20) |  | 1.16 (1.05–1.29) |  |
| Sex | Female | - | - | - | <0.001 |
|  | Male | 1.41 (1.30–1.53) |  | 1.22 (1.09–1.36) |  |
| Disease | Breast | Ref | <0.001 | Ref | <0.001 |
|  | Endocrine | 1.00 (0.75–1.31) |  | 0.74 (0.49–1.07) |  |
|  | Gastrointestinal | 1.24 (1.06–1.44) |  | 0.88 (0.73–1.09) |  |
|  | Gynecological | 0.98 (0.83–1.16) |  | 0.71 (0.57–0.90) |  |
|  | Hematological | 2.30 (1.91–2.77) |  | 2.24 (1.79–2.82) |  |
|  | Head and neck | 1.84 (1.54–2.20) |  | 0.78 (0.60–1.02) |  |
|  | Male genitals | 0.65 (0.49–0.85) |  | 0.60 (0.40–0.85) |  |
|  | Mesothelial and soft tissue | 2.10 (1.55–2.80) |  | 1.77 (1.21–2.55) |  |
|  | Other | 5.92 (4.54–7.74) |  | 7.58 (5.68–10.12) |  |
|  | Respiratory and intrathoracic organs | 4.32 (3.65–5.13) |  | 3.30 (2.69–4.07) |  |
|  | Skin | 1.69 (1.29–2.20) |  | 1.98 (1.44–2.71) |  |
|  | Urinary tract | 0.79 (0.59–1.04) |  | 0.76 (0.51–1.08) |  |
| Stage | 1 | Ref |  | Ref |  |
|  | 2 | 1.46 (1.21–1.76) |  | 1.58 (1.21–2.08) |  |
|  | 3 | 2.03 (1.71–2.42) |  | 1.83 (1.42–2.37) |  |
|  | 4 | 4.43 (3.78–5.22) |  | 4.34 (3.46–5.50) |  |
| Pain | No |  | <0.001 |  | <0.001 |
|  | Yes | 2.65 (2.44–2.90) |  | 2.00 (1.80–2.24) |  |
| Dyspnea | No |  | <0.001 |  | <0.001 |
|  | Yes | 5.09 (4.63–5.60) |  | 3.91 (3.49–4.38) |  |
| Edema | No |  | <0.001 |  | <0.001 |
|  | Yes | 2.77 (2.40–3.18) |  | 2.78 (2.36–3.26) |  |
| Delirium | No |  | <0.001 |  | <0.001 |
|  | Yes | 4.84 (3.15–7.53) |  | 6.32 (4.09–9.70) |  |
| Xerostomia | No |  | <0.001 |  | <0.001 |
|  | Yes | 3.41 (2.61–4.46) |  | 4.61 (3.48–6.07) |  |
| Ascites | No |  |  |  | <0.001 |
|  | Yes | 1.12 (0.98–1.27) |  | 1.20 (1.01–1.42) |  |
| Dysphagia | No |  | <0.001 |  | <0.001 |
|  | Yes | 2.29 (1.99–2.62) |  | 0.78 (0.62–0.98) |  |
| Anorexia | No |  | <0.001 |  | <0.001 |
|  | Yes | 2.88 (2.55–3.26) |  | 2.44 (2.10–2.83) |  |
